# A Multi-Omics Analysis of Metastatic Melanoma Identifies a Germinal Center-Like Tumor Microenvironment in HLA-DR-Positive Tumor Areas

**DOI:** 10.3389/fonc.2021.636057

**Published:** 2021-03-25

**Authors:** Laura Gadeyne, Yannick Van Herck, Giorgia Milli, Zeynep Kalender Atak, Maddalena Maria Bolognesi, Jasper Wouters, Lukas Marcelis, Angeliki Minia, Vaia Pliaka, Jan Roznac, Leonidas G. Alexopoulos, Giorgio Cattoretti, Oliver Bechter, Joost Van Den Oord, Frederik De Smet, Asier Antoranz, Francesca Maria Bosisio

**Affiliations:** ^1^ Department of Pathology, UZ Leuven, Leuven, Belgium; ^2^ Department of General Medical Oncology, University Hospitals Leuven, Leuven, Belgium; ^3^ Translational Cell and Tissue Research, Department of Imaging and Pathology, KU Leuven, Leuven, Belgium; ^4^ Laboratory of Computational Biology, KU Leuven, Leuven, Belgium; ^5^ Pathology, Department of Medicine & Surgery, University of Milano-Bicocca, Milan, Italy; ^6^ ProtATonce Ltd, Athens, Greece; ^7^ Life Sciences Research Unit, University of Luxembourg, Belvaux, Luxembourg; ^8^ Biomedical Systems Laboratory, Department of Mechanical Engineering, National Technical University of Athens, Athens, Greece

**Keywords:** melanoma, single-cell, multi-omics, multiplex, HLA-DR

## Abstract

The emergence of immune checkpoint inhibitors has dramatically changed the therapeutic landscape for patients with advanced melanoma. However, relatively low response rates and a high incidence of severe immune-related adverse events have prompted the search for predictive biomarkers. A positive predictive value has been attributed to the aberrant expression of Human Leukocyte Antigen-DR (HLA-DR) by melanoma cells, but it remains unknown why this is the case. In this study, we have examined the microenvironment of HLA-DR positive metastatic melanoma samples using a multi-omics approach. First, using spatial, single-cell mapping by multiplexed immunohistochemistry, we found that the microenvironment of HLA-DR positive melanoma regions was enriched by professional antigen presenting cells, including classical dendritic cells and macrophages, while a more general cytotoxic T cell exhaustion phenotype was present in these regions. In parallel, transcriptomic analysis on micro dissected tissue from HLA-DR positive and HLA-DR negative areas showed increased IFNγ signaling, enhanced leukocyte adhesion and mononuclear cell proliferation in HLA-DR positive areas. Finally, multiplexed cytokine profiling identified an increased expression of germinal center cytokines CXCL12, CXCL13 and CCL19 in HLA-DR positive metastatic lesions, which, together with IFNγ and IL4 could serve as biomarkers to discriminate tumor samples containing HLA-DR overexpressing tumor cells from HLA-DR negative samples. Overall, this suggests that HLA-DR positive areas in melanoma attract the anti-tumor immune cell infiltration by creating a dystrophic germinal center-like microenvironment where an enhanced antigen presentation leads to an exhausted microenvironment, nevertheless representing a fertile ground for a better efficacy of anti-PD-1 inhibitors due to simultaneous higher levels of PD-1 in the immune cells and PD-L1 in the HLA-DR positive melanoma cells.

## Introduction

Primary Cutaneous Melanoma (PCM) is characterized by an aggressive course including metastatic spread directly proportional to the depth of invasion of the tumor cells into the skin (typically defined as the Breslow thickness) (1). In addition, PCM is also characterized by one of the highest somatic mutation rates (2), from which only a minority are driver mutations, while rest are passenger mutations that do not play a role in tumor development or progression. Yet, this high tumor mutational burden (TMB) can translate into a high amount of neo-antigens available for antigen recognition by the immune system, a feature that has been attributed to the strong immunogenicity of melanoma and the clinical effect of immunotherapy in these patients. Indeed, immune checkpoint inhibitor (ICI) therapy aimed at blocking the PD-1/PD-L1 axis was approved by the FDA in 2014 for the treatment of PCM and is now being used as a standard of care for patients with irresectable stage III or stage IV disease, and as adjuvant therapy in stage III melanoma (3). Unfortunately, ICI response rates are still relatively low, at least when given in monotherapy, and a significant percentage of patients suffers from severe, immune-related adverse events, which in rare cases can even be fatal (4–6). Therefore a great demand exists for predictive biomarkers to allow a better patient selection before exposing them to ineffective, potentially toxic therapies, for which a number of markers have already been proposed. For example, the density of CD8+ T cells in both the border and bulk of the tumor have been correlated with a higher response to ICI (7, 8). Likewise, the presence of a high TMB, an interferon-gamma (IFNγ)-related mRNA profile and a T cell-inflamed gene expression profile have also been proven to have a positive predictive value (9–12). Interestingly, the expression of Human Leukocyte Antigen-DR (HLA-DR), which is a Major Histocompatibility Complex (MHC) class II molecule, has also been associated with outcome in ICI-treated melanoma patients (13–16). Nevertheless, despite the plethora of different markers, none of those mentioned can predict response with acceptable accuracy and none of them have been prospectively evaluated in the context of a clinical trial which is why they have not found their way to daily clinical practice.

HLA-DR molecules are dimeric surface receptors that are mainly expressed in professional antigen presenting cells to present antigen peptides to CD4+ T cells in order to elicit an adaptive immune response. HLA-DR, HLA-DQ and HLA-DP are the three major MHC class II genes. Among these, HLA-DR is the most ubiquitously expressed. Expression of HLA-DR molecules requires the expression of CIITA, a transcriptional coactivator known as the master regulator of MHC II transcription. In an inflammatory microenvironment, MHC II molecules can be aberrantly expressed by non-hematopoietic cells, including melanoma cells (17), which, similar to PD-L1 expression in melanoma, can occur following secretion of IFNγ by NK cells and cytotoxic T cells (18, 19). Binding of IFNγ to its receptor induces JAK/STAT signaling, which initiates transcription of CIITA *via* binding of STAT-1 to the CIITA promoter IV (20). Intuitively, it could therefore be hypothesized that the aberrant MHC II expression by melanoma cells would stimulate the immune response by increasing the presentation of tumor-specific antigens. However, other interactions are needed to elicit T cell activation, in particular the expression of co-stimulatory receptors (21, 22). Contrary to that, MHC II is also a ligand to Lymphocyte-activation gene 3 (LAG3), a checkpoint molecule that is expressed by activated T lymphocytes. Upon sustained interaction with MHC II positive melanoma cells, activated lymphocytes will evolve into exhaustion, and thus become inactivated (23). These different mechanisms and the additional cofactors may explain why MHC II expression is associated with an unfavorable prognosis in some studies, but with tumor regression and longer survival in others (18, 24–28).

**Figure 1:**
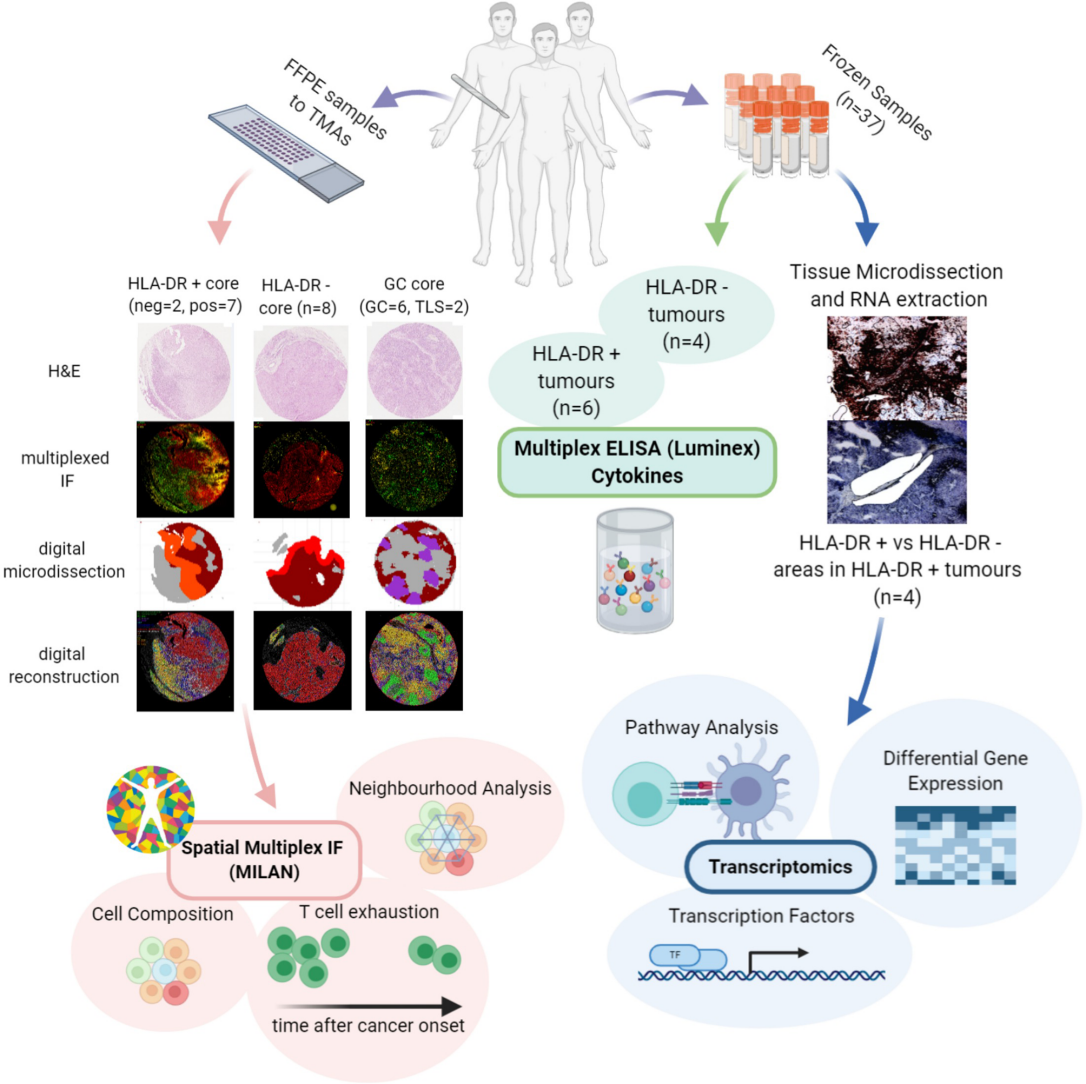
Investigation of the microenvironment of HLA-DR positive metastatic melanoma samples using multi-omics approach. FFPE samples from patients expressing HLA-DR+ metastatic melanoma were selected and transferred to TMAs. Then, MILAN was performed on HLA-DR+ and HLA-DR- areas in order to characterize the immune microenvironment at single-cell level. The data extracted allowed investigating the cell composition, performing the neighborhood analysis and revealed presence of T cell exhaustion. The cell composition and neighborhood analyses was repeated for germinal centers/tertiary lymphoid structures to provide more evidence about the similarity between the features characterizing the immune microenvironment of HLA-DR + and HLA-DR- areas and the germinal centers. In parallel, Multiplexed immunohistochemistry was applied to the frozen samples of HLA-DR+ tumors to reveal HLA-DR+ regions. The regions were micro dissected to compare Pos and NegInPos areas of selected cases with adequate RNA quality. Gene expression analysis, transcription activity and pathway analysis revealed signatures related to both pro-inflammatory and anti-inflammatory roles and an upregulation of multiple biological pathways primarily involving the immune system function. Finally, multiplexed ELISA was performed on HLA-DR+ and HLA-DR- tumors taken from the frozen sample dataset to investigate which cytokines predominantly drive the composition of the tumour microenvironment.

Although some features such as enhanced tumor infiltrating lymphocytes (TILs), the presence of a lymphocytic activation pathway and the occurrence of tumor-specific CD4+ T cells preventing the activation of cytotoxic T cells through production of tumor necrosis factor α (TNFα) have been reported in HLA-DR+ melanoma (13, 29, 30), relatively little is known about the underlying mechanisms and the actual composition of the tumor microenvironment in these areas. The goal of our study was therefore to explore the tumor microenvironment in HLA-DR positive areas of malignant melanoma in order to get deeper insights into the composition of the infiltrate and the possible interactions between the local inflammatory cells. To this aim, we characterized the immune microenvironment in HLA-DR positive and negative areas using a multi-omics approach, combining spatial single-cell profiling using multiplexed immunohistochemistry, but also RNA sequencing (RNA-seq) from micro dissected material and cytokine profiling (**Figure 1**). As such, we identified in HLA-DR positive tumors a concentration of immune cells specifically in HLA-DR-expressing areas of the tumor, and this was due to a germinal center-like microenvironment. We found evidence at multiple levels that this microenvironment was also characterized by T cell exhaustion, hyperactivity of the antigen presentation pathways, and simultaneous higher levels of PD-1 in the immune cells and PD-L1 in the HLA-DR+ melanoma cells.

## Materials and Methods

### Patient Selection

The clinical features of the patients included in the study are summed up in **Supplementary Table 1**. A first data set of 9 melanoma metastases (4 HLA-DR+, 5 HLA-DR-), 4 lymph nodes from completion lymphadenectomies and 1 case with tertiary lymphoid structures adjacent to a cutaneous melanoma metastasis with available FFPE material were collected from the archive of the Department of Pathology of the UZ Leuven (Leuven, Belgium) and assembled in a Tissue Micro Array (TMA) with a variable number (1–3) of 2-mm cores per patient according to the size of the tumor and the extension of the HLA-DR+ areas in order to achieve a satisfactory representation of the HLA-DR+ and HLA-DR- areas. A second data set of 37 fresh frozen melanoma metastases was collected in order to select cases for laser microdissection and NGS sequencing. HLA-DR immunohistochemistry (Abcam, SPM289, 1:1000, 4 µ slides, targeting the alpha subunit of HLA-DR molecule) was performed, and 4 HLA-DR+ cases were selected for microdissection on the basis of a higher RNA quality obtained after RNA extraction. Other 10 frozen samples (6 HLA-DR+ and 4 HLA-DR-) from this data set were used as a validation cohort for multiplex ELISA analysis.

### Multiplexed Immunohistochemistry Using the MILAN Method

Multiplexed immunofluorescence staining was performed according to the previously published MILAN protocol (31, 32), which makes use of a cyclic staining-stripping approach. An overview of the panel of markers and antibodies used can be found in **Supplementary Table 2**. Immunofluorescence images were scanned using the NanoZoomer S60 Digital slide scanner (Hamamatsu, Japan) at 20X objective with resolution of 0,45 micron/pixel. Image analysis, feature extraction and phenotypic identification of the main cell types was performed following the procedure described in Bosisio et al. (33). Briefly, DAPI images from consecutive rounds were aligned (registered) using the Turboreg and MultiStackReg plugins from Fiji/ImageJ (version 1.51 u). The coordinates of the registration were saved as Landmarks and applied to the rest of the channels. Tissue autofluorescence was subtracted from an acquired image in a dedicated channel, for FITC, TRITC and Pacific Orange. The TMA was segmented into tissue cores using a custom macro. Cell segmentation, and feature extraction were performed using a custom pipeline in CellProfiler (version 2014-07-23T17:45:00 6c2d896). MFIs were further normalized to Z-scores as recommended in Caicedo JC et al. (34). Z-scores were trimmed between −5 and +5 to avoid a strong influence of any possible outliers in the downstream analysis. Cell subpopulations were identified by applying in a subset of all cells (25,000) three different clustering methods: PhenoGraph, ClusterX and K-means over the 38 included phenotypic markers: CD138, CD14, CD141, CD16, CD163, CD1A, CD1C, CD2, CD20, CD21, CD23, CD248, CD25, CD27, CD3, CD303, CD31, CD34, CD4, CD5, CD56, CD64, Cd68, CD79A, CD8, CK, FOXP3, GRB7, HLA-DR, IRF4, IRF8, LYZ, MELANA, PAX5, PNAD, PODOPLANIN, PRDM1, and S100B. A fingerprint for each cluster was constructed by averaging the expression of all their cells for each marker. These fingerprints were associated with known cell phenotypes by manual annotation from domain experts (FMB, YVH). This way we have three annotations for each cell, one per clustering method. The final annotation was obtained by applying a consensus-based approach: if two or more of the clustering methods agreed on the assigned phenotype, then the cell was labelled as such. If all three clustering methods assigned different cell phenotypes, the cells were labelled as “other”. In **Supplementary Figure 1A** are shown the fingerprints with the expression of all the markers used for clustering in relation to every identified cell type *via* the consensus-based approach. These fingerprints were used to label the cell phenotype of the remaining cells in the entire dataset (minimum of Euclidean distance). We further characterized specific cell types by applying manual gating to the expression (asinh transformed) of specific markers, as indicated in **Supplementary Figures 1B–D**. We identified T Follicular Helpers based on PD-1 expression in the T helpers (Th) cluster (TFH, PD-1 high); based on expression of BCL6 and BCL2, B cells were sub classified into germinal center B cells (BCL6+/BCL2-, BC_GerminalCenter), early germinal center B cells (BCL6+/BCL2+, BC_EarlyGerminalCenter) and B cells not further specified (BCL6-/BCL2- and BCL6-/BCL2+, BC); finally, melanoma cells were stratified into HLA-DR+ and HLA-DR- melanoma cells (HLADRpos_mel and HLADRneg_mel).

### In Silico Tissue Microdissection

We digitally micro dissected the tissue cores by fragmenting the tissue into 50x50 pixel tiles (~22 sq micrometers). Tiles with at least 1 cell identified as tumor were initially defined as tumor areas. To reduce the impact of potential outliers a median filter was applied to the obtained tumor masks. Similarly, to define germinal centers we created a mask for the tiles containing at least 50% of follicular dendritic cells (fDC), germinal center B cells (BC_GerminalCenter) or B cells not further specified (BC) in the tile. Then, we filtered out all the objects in the mask smaller than 10 tiles (~220 sq micrometers). Finally, we removed those objects not containing all three cell types used to define the mask (fDC, BC_GerminalCenter, BC).

To reproduce as much as possible the conditions that we would have applied during real life microdissection, in a second step we manually dissected the tumor areas of HLA-DR+ tumors into HLA-DR+ areas (“Pos”) and HLA-DR- areas (“NegInPos”). Given that HLA-DR+ areas were always in the tumor edge, only HLA-DR- areas at the tumor border were included. In addition, we manually micro dissected the tumor borders of HLA-DR- tumors (“NegTum”). Finally, we micro dissected germinal centers from reactive lymph nodes (“GC”) and germinal centers from tertiary lymphoid structures (“TLS”) from a cutaneous melanoma metastasis.

### Cell Composition Analysis

We compared the cell proportion and density (cell counts per square millimeter) of the different micro dissected areas using Wilcoxon’s rank sum test. The reader should note that we removed the “other”, “stroma”, and “epithelial” cell phenotypes from the comparison due to the lack of relevance of these cell types for our analysis. An overview of the p-values derived from all these comparisons can be found in **Supplementary Table 4**. P-values were not adjusted for multiple comparisons due to the relatively low number of samples and the exploratory nature of the study.

### Neighborhood Analysis

We characterized the immune landscape of the different dissected tissue types by neighborhood analysis (35). We focused on short distance cell-cell interactions by selecting a kernel of radius = 50px (~22 micrometers) and assigned an empirical p-value by a permutation test (N = 1000). The size of the kernel that defines the neighborhood of a cell is a user-defined parameter and depends on whether we want to see short/medium/long-distance interactions. For this particular study, we are interested in short-distance interactions and have set the radius of the neighborhood kernel to 50 pixels (~22 micrometers). Considering that the average cell-radius size in this dataset is of 7 micrometers and that the distance between two cells is calculated from their centers, this corresponds to less than 1 cell diameter from the edge of the cell. In brief, the neighborhood analysis method described by Schapiro et al. (35) counts specific cell pairs at a user-defined distance and compares them with the counts that could be found in the random case. This random case is built by permuting the labels of all the cells a number of times (N=1000). This approach allows us to compare the number of interactions observed in the real tissue and compare them with randomized cases to assign a significance value to a cell-cell interaction representative of the spatial organization of the cells. Neighborhood analysis was limited to the in-silico micro dissected areas (Pos, NegInPos, NegTum, GC and TLS). In the tumor areas (Pos, NegInPos and NegTum), the large majority of cells are melanoma cells. Therefore, we did not randomize the position of melanoma cells in the permutations since the melanoma cells are organized in large clusters with relatively few interactions to the rest of the cells. A complete randomization would thus exaggerate all the other cell-cell interactions which can lead to misleading results. Interaction scores across different samples were integrated using a weighted average. The weight for each sample was defined as the log10 of the geometric average of the counts for the two cell types being considered. Finally we classified the nature of the interaction between two cells types into “strong interaction” if the number of counts in the observed tissue was higher than 950 random cases (p-value < 0.05), “moderate interaction” if the number of counts in the observed tissue was higher than 900 random cases (p-value < 0.1), and “no interaction” otherwise (p-value > 0.1). “Other”, “stroma”, and “epithelial” cell phenotypes were not included in the neighborhood analysis due to the lack of relevance of these cell types.

### Laser Capture Microdissection and RNA Sequencing

**Figure 2:**
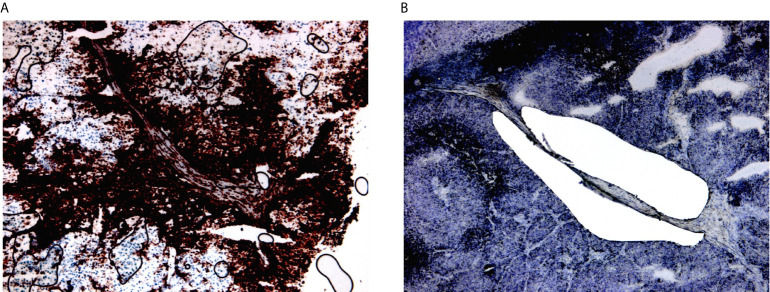
Laser microdissection of HLA-DR+ areas in HLA-DR+ tumors. **(A)** HLA-DR+ areas were identified using conventional immunohistochemical staining for HLA-DR. HLA-DR expression in melanoma cells located at the margin of the tumors at margins of the tumour nodules, at the so-called tumour-stromal interface. **(B)** Laser microdissection was executed on the immediately consecutive section after morphological recognition of the HLA-DR+ region.

HLA-DR+ tumor/areas were identified by screening all the mentioned data sets *via* conventional immunohistochemical staining for HLA-DR (Abcam, SPM289, 1:1000). A tumor was considered positive if showing tumor areas with HLA-DR expression in melanoma cells. HLA-DR expression in our data sets was generally zonal, as expected, and located at the margin of the tumors at the tumor-stroma interface. Two expert dermatopathologists (LG, FB) evaluated the HLA-DR positivity and classified the tumors as positive, distinguishing HLA-DR expression in inflammatory cells (e.g. macrophages/dendritic cells) from real expression in melanoma cells in a similar way as it is done in the clinics for PD-L1 evaluation, that is considered to be the gold standard. In this way, only areas with real HLA-DR expression in melanoma cells (and not exclusively in inflammatory cells) were microdissected. Laser microdissection was executed by the expert dermatopathologists on the section immediately consecutive to the one that was stained for HLA-DR (**Figure 2**). Laser microdissection (LMD) of HLA-DR+ and HLA-DR- tumor areas was performed in HLA-DR+ tumors. The microdissection was restricted to the marginal zone of the tumors both in positive and negative areas limiting the amount of stroma included to strictly peri-tumor. An average of around 2000 tumor cells was dissected per vial. RNA extraction from the LMD samples was performed by usage of a special RNA extraction kit for LMD samples (RNAqueous^®^-Micro Kit, Life Technologies Corporation). Before submission to RNA sequencing analysis, RNA quality of the LMD samples was assessed using the Bioanalyzer RNA 6000 pico assay (Agilent). Four cases with acceptable RNA Integrity Number (RIN between 3,90-6,90) were selected. RNA sequencing analysis was performed using the Quantseq protocol (Lexogen).

### Computational Analysis of Gene Expression Data

RNA-Seq.fastq files were aligned to the reference genome (CRCh38.p12, gencode.v31) using STAR v2.7. Raw counts were then obtained using the featureCounts function from the RSubread R package. Counts were normalized using the DESeq2 R package. The sequencing data is available at the European Nucleotide Archive (ENA) under the accession number PRJEB41749. Next, samples were grouped and we compared the expression of HLA-DR+ vs HLA-DR- areas in matched patients. For differential gene expression analysis we applied the DESeq2 R package with standard thresholds (p-value = 0.05, logFC = ± 1). Since transcription factors might only become active in a phosphorylated state or in the presence of coactivators, differential mRNA expression cannot provide us with information about their activation. Therefore, the activity of the transcription factors was predicted based on the expression of their targets using DOROTHEA (36). Only transcription factors with a confidence level of A, B, and C were kept for this analysis. Because complex and heterogeneous phenotypes are often not the answer to large changes in individual genes but rather smaller changes in functionally correlated genes, we subsequently performed pathway analysis using Piano (37). Implementing the Piano framework, we used the following 10 pathway analysis methods and gene-level-statistics: Fisher (p-value), Stouffer (p-value), Reporter (p-value), tailStrength (p-value), Page (t-value), GSEA (t-value), maxmean (t-value), Mean (FC), Median (FC), Sum (FC). The molecular Signatures Database (MSigDB), curated pathways (c2), canonical pathways (cp) version 7.0 was used for the definition of the gene sets. This database contains 11763 genes mapping to 2199 pathways.

### Luminex Analysis

Proteins were extracted from five 10 micrometers-thick cryostat sections according to the protocol of Allred et al. (38). We built and validated a customized Multiplex ELISA panel for the Luminex Flexmap 3D at Protavio (Athens, Greece), coupling different magnetic beads from Luminex with the capture antibody of the duoset ELISA from R&D Systems and Standard ABTS ELISA Development Kit from Peprotech against human INF*γ*, IL6, IL10, TNFα, IL4, CXCL10, IL17, IL13, CCL18, TGFβ, IL23, CXCL13, CXCL12, and CCL19. Initially, we explored the information content of these 14 markers using unsupervised dimensionality reduction (hierarchical clustering and uMap). Next, we trained machine-learning models (linear discriminant analysis, LDA) using panels of 1 to 14 markers at the time. We fitted each model following a leave-one-out cross-validation scheme. Mean Fluorescence Intensities (MFIs) were normalized (z-scores) before training the LDA models. For each panel size, the best panel was selected as the one maximizing accuracy. Models with the same accuracy were prioritized by minimizing the residual probabilities. Following Ockham’s razor, we want the simplest model that explains the data. To that end, we finally selected the optimal model by applying the elbow criterion on the generated Pareto front to select the smallest panel size that provides a good predictive ability.

## Results

### Single Cell Characterization of the Tumor Microenvironment of HLA-DR Positive and Negative Areas in Metastatic Melanoma

**Figure 3:**
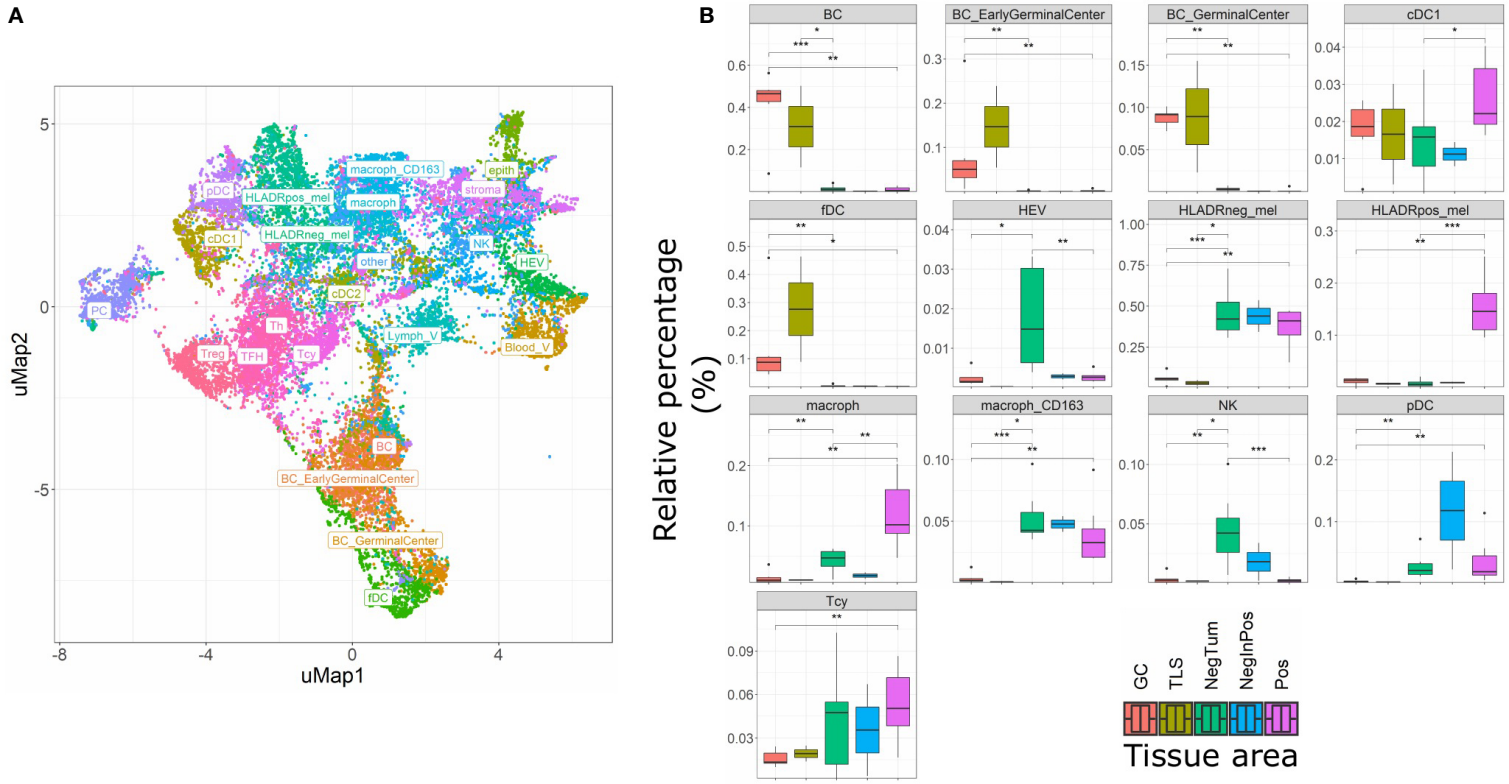
Single cell composition of HLA-DR+ areas, HLA-DR- areas in positive tumors, HLA-DR- tumors, germinal centers and tertiary lymphoid structures. **(A)** uMap of a subset of cells (22,000) from the complete dataset included in this analysis (544,910 cells). BC= B cell not further specified; BC_EarlyGerminalCenter= Early germinal center B cell; BC_GerminalCenter= Germinal center B cell; cDC1 = Classical dendritic cell type I; fDC= Follicular dendritic cell; HEV= High endothelial venule; HLAneg_mel= HLA-DR negative melanoma cell; HLADRpos_mel= HLA-DR positive melanoma cell; macrop= Macrophage; macrop_CD163= CD163 positive macrophage; NK= Natural Killer cell; pDC= Plasmacytoid dendritic cell; Tcy= Cytotoxic T cell; Treg= Regulatory T cell; TFH = T Follicular Helper cells; Th= T helper cell; PC = Plasma cell; cDC2 = Classical dendritic cell type II; Stroma= Stromal cell; epith= Epithelial cell; Lymph_V= Lymphatic vessel; other= Cells not further specified. **(B)** Boxplots indicating the relative proportion of different cell-types in the different micro dissected areas, from left to right: ‘GC’ (Germinal center from reactive lymph nodes; orange), TLS (germinal centers from tertiary lymphoid structures, ochre), ‘NegTum’ (Tumour border of HLA-DR negative tumors, green), ‘NeginPos’ (HLA-DR negative area of HLA-DR positive tumors, blue) and ‘Pos’ (HLA-DR positive area of HLA-DR positive tumors, pink). Significance levels indicate: *p-value ≤ 0.05, **p-value ≤ 0.01, ***p-value ≤ 0.001. P-values are derived from Wilcoxon tests (not fdr corrected).

The main goal of this study was aimed at defining the composition and characteristics of the tumor microenvironment of HLA-DR+ metastatic melanoma samples compared to HLA-DR- negative areas/tumors at single cell and spatial level. As a first step, we screened a cohort of metastatic melanoma tissue samples for HLA-DR expression. By performing IHC for HLA-DR in these samples, we identified samples in which the melanoma cells did not express HLA-DR (HLA-DR-) and samples in which tumor cells were expressing high levels of HLA-DR (HLA-DR+) (**Supplementary Table 1**). Importantly, in this second group, HLA-DR expression in the tumor cells was mostly not homogeneous but expressed mainly at the borders of the tumor where tumor cells were interacting with the adjacent stromal tissue. The borders of the HLA-DR+ tumors were not circumferentially positive but positive areas and negative areas could both be present at the borders of a HLA-DR+ tissue sample (**Figure 1**). Therefore, in HLA-DR+ tumors we analyzed and compared the microenvironment of HLA-DR+ areas (“Pos”) and HLA-DR- areas (“NegInPos”) and in HLA-DR- tumors we sampled the border zone (“NegTum”). As the next step, we wished to understand the cellular composition of each of these regions, with a strong focus on the immune infiltrates and their interactions. To achieve this, we performed spatially-resolved, single-cell, multiplexed immunohistochemistry using the MILAN method (see methods) using a broad panel of inflammatory, tumor and other stromal cell markers (**Supplementary Table 2**). Following quality control and cell clustering of ~544k DAPI+ cells using the main phenotypic markers across the included samples, the large majority could be unequivocally mapped and identified as tumor, endothelial, myeloid (macrophage or dendritic cells), T, B, NK, and stromal cells (**Supplementary Figure 1A**). These were subsequently combined with a number of functional markers, resulting in the identification of 23 robust cell types (**Figure 3A**, **Table 1**). Using this approach, we observed that ~7% of the identified MelanA+/S100B+ melanoma cells were also positive for HLA-DR (13497 HLA-DR+ vs 177829 HLA-DR- cells). Areas enriched in HLA-DR+ melanoma cells (“Pos”) showed the same zonal distribution as described for the conventional immunohistochemically staining for HLA-DR (**Figure 2**). In each of the samples, we subsequently defined the different areas (“Pos”, “NegInPos” and “NegTum”), including also non-tumor lymphoid areas (Germinal centers “GC” and tertiary lymphoid structures “TLS”) for comparison. Next, we determined both the relative distribution as well as the cell density of the identified cell types across these different areas (**Figure 3B**, **Supplementary Figures 2–3** and **Supplementary Table 3**). In the lymphoid compartment, Tcy were generally more present in tumor than GC/TLS, with a significant difference between Pos and GC, while Th, Treg did not show significant differences. All subtypes of B cells were enriched as expected in GC/TLS compared to the tumor. NK cells were strongly enriched in NegTum compared to HLA-DR+ tumors. Among dendritic cells, the main differences were the expected high abundance of fDC in GC; an enrichment of cDC1 in Pos compared to both the NegTum and NegInPos; a higher density, but not proportion, of cDC1 in GC in comparison with Neg; and a general enrichment of pDC in the tumor compared to GC. The macrophage compartment showed a peculiar distribution among the different areas: M1-like macrophages were abundantly present in Pos compared to NegTum, while NegInPos had a lower proportion of them (though this difference was non-significant for density). M2-like macrophages, on the other hand, were entirely absent in the GC/TLS areas, while they were present in all tumor areas. We found differences also in the vascular composition of the areas: high endothelial venules (HEV) were significantly more present in NegTum compared to GC/TLS or HLA-DR+ tumors, in which HEVs were equally less represented in both Pos and NegInPos. There were also some trends that did not reach significance but for which borderline p-values were observed. In particular, we found an enrichment of plasma cells in Pos compared to the adjacent NegInPos, of lymph vessels in NegInPos and of blood vessels in the tumor areas, while Pos tended to have less TFH than NegTum and GC/TLS. **Supplementary Table 4** includes the p-values for all comparisons in terms of cell density as well as cell proportion.

**Table 1:** Main cell types identified with MILAN.

Cell type	Cell subtype	Abbreviation	# cells	% overall	% subtype	Cell density(cells/mm²)
B cell	Not further specified	BC	39 600	7.27	61.09	13 356
	Early germinal center	BC_EarlyGerminalCenter	6 920	1.27	10.67	2 334
	Germinal center	BC_GerminalCenter	7 161	1.31	11.05	2 415
Plasma cell	N/A	PC	11 146	2.05	17.19	3 759
T cell	T helper	Th	38 308	7.03	41.34	12 920
	Regulatory T cell	Treg	13 074	2.4	14.11	4 410
	T Follicular Helper	TFH	16 845	3.09	18.18	5 681
	Cytotoxic T cell	Tcy	24 432	4.48	26.37	8 240
Natural Killer cell	N/A	NK	7 581	1.39	100	2 557
Dendritic cell	Classical dendritic cell type I	cDC1	22 736	4.17	47.14	7 668
	Classical dendritic cell type II	cDC2	11 841	2.17	24.55	3 994
	Follicular dendritic cell	fDC	5 325	0.98	11.04	1 796
	Plasmacytoid dendritic cell	pDC	8 329	1.53	17.27	2 809
Macrophage	Macrophage	Macroph	23 776	4.36	49.46	8 019
	CD163 positive macrophage	Macroph_CD163	24 291	4.46	50.54	8 193
Melanoma	HLA-DR+ melanoma	HLADRpos_mel	13 497	2.48	7.05	4 552
	HLA-DR- melanoma	HLADRneg_mel	177 829	32.63	92.95	59 977
Vasculature	Blood vessel	Blood_V	16 388	3.01	44.77	5 527
	High Endothelial Venule	HEV	6 460	1.19	17.65	2 179
	Lymphatic vessel	Lymph_V	13 757	2.52	37.58	4 640
Epithelial cell	N/A	Epith	1 941	0.36	100	655
Stromal cells	N/A	Stroma	10 546	1.94	100	3 557
Other	N/A	other	43 127	7.91	100	14 546

Overview of the number of cells detected and both the relative proportion (% of all cells and the % of each subtype) as well as the cell density (cells/mm²) of all identified cell types in the MILAN analysis, not discriminating between the different micro dissected areas. For the expression/marker profile of each cell type, we refer to **Supplementary Figure 1**. N/A, not applicable.

**Figure 4:**
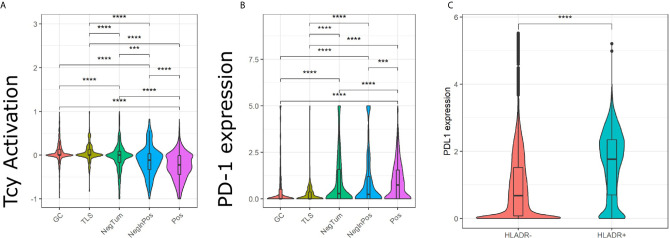
Analysis of Tcy activation status, TIM3 and PD-1 in the *in-silico* micro dissected areas. Violin plots of the activation score **(A)** and PD-1 **(B)** for the cytotoxic T cells (Tcys) in the different micro dissected areas. P-values are derived from pairwise t-test **(A)** and Wilcoxon test **(B)**. **(C)** Difference in expression of PD-L1 in HLA-DR+ melanoma versus HLA-DR- melanoma. P-values are derived from Wilcoxon Rank Sum and Signed Rank Test. Significance levels indicate: *** p-value ≤ 0.001, **** p-value ≤ 0.0001. P-values were adjusted using the false discovery rate (fdr) method.

Next, we evaluated the activation status of the Tcy located in the different areas according to a defined algorithm that makes use of a panel of activation and exhaustion markers including CD69, TIM3, OX40, LAG3 as previously published (**Figure 4A**) (33). We compared activation levels in the different areas using a t-test with false-discovery-rate (fdr) correction. We found levels of exhaustion of the Tcy to be higher in HLA-DR+ tumors compared to the HLA-DR- ones (p-adj=5.80×10^-61^), and within the positive tumors, the Tcy were particularly exhausted in Pos compared to NegInPos (p-adj=9.92×10^-7^). Moreover, since HLA-DR overexpression was found to be associated with response to anti-PD-1 therapy (13, 16), we also evaluated the expression of PD-1 in the different areas, thereby observing that Pos also had higher levels of PD-1 expression (Wilcoxon rank sum test, fdr corrected, p-adj_NegTum_=4.22×10^-7^, p-adj_NegInPos_=3.54×10^-4^) (**Figure 4B**). In addition, considering higher PD-L1 expression being described in HLA-DR+ melanoma cell lines and that HLA-DR mediated signaling increases the expression of PD-L1 in melanoma cells (13, 39), we compared the expression of PD-L1 between HLA-DR- and HLA-DR+ melanoma cells and could observe significant higher expression levels in the HLA-DR+ melanoma cells (Wilcoxon rank sum test, fdr corrected, p-adj < 1x e^-16,^ **Figure 4C**). Summarizing the above, we could show simultaneous higher PD-1 expression on the immune cells surrounding the HLA-DR+ melanoma cells that have higher PD-L1 expression compared to the HLA-DR- melanoma cells.

**Figure 5:**
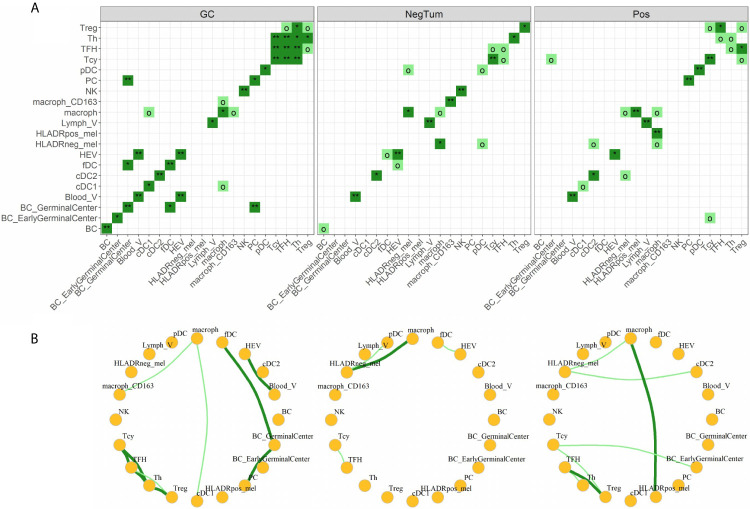
Neighborhood analysis. **(A)** Heat maps indicating the significance of cell-cell interactions in the different micro dissected areas. Interactions go from “strong interaction” (dark green, p-value < 0.05), “moderate interaction” (light green, p-value < 0.1), to “no interaction” (white, p-value ≥ 0.1). Significance levels indicate: o p-value < 0.1, *p-value < 0.05, **p-value < 0.01. **(B)** Social networks obtained from moderate and strong interactions. Each node in the graph represents a cell-type and each edge an interaction.

Finally, we investigated the various cell-cell interactions between all the identified cellular subtypes in each of the defined areas (**Figures 5A, B**). As positive control, we found that the GC had a florid interaction network, including the ones that we expected in the B cell (where the various B cell and plasma cells are interacting with the fDCs cells) and T cell zones (with a network of TFH, Th, Tcy and Treg cells) (**Figures 5A, B**, left panels). In the tumor areas, we mainly found interactions of M1-like macrophages with either HLA-DR+ and HLA-DR- melanoma cells. Though, the strength of interaction and the other actors involved in the interactions with the two melanoma cell types were different in positive and negative tumors: while in NegTum HLA-DR- melanoma cells strongly interacted with M1-like macrophages and also had interactions with pDC (**Figures 5A, B**, central panels), in Pos the interaction between HLA-DR- melanoma cells and M1-like macrophages was weaker and accompanied by interactions with cDC2, while the stronger interaction was between M1-like macrophages and HLA-DR+ melanoma cells (**Figures 5A, B**, right panels). In addition, specifically in Pos, small communities of mixed T and B cells were found, where in particular Treg interacted with TFH and Tcy, TFH with Th, and Tcy with BC_GerminalCenter. HLA-DR negative tumors presented instead a smaller T community composed of Tcy in contact with TFH.

### Gene Expression Analysis, Transcription Factor Activity Analysis and Pathway Analysis Identify Signatures Related to a Mixed B-T Microenvironment, With Upregulation of IFNγ and Antigen Presentation but Also With Clues Towards Immunosuppression in HLA-DR+ Tumor Areas

**Figure 6:**
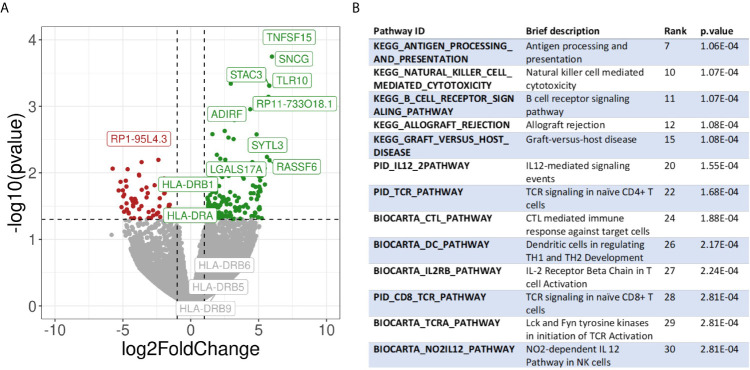
Transcriptomic analysis. **(A)** Volcano plot showing differential gene expression between HLA-DR+ and HLA-DR- tumour areas. The x-axis represents the log2 of the fold change while the y-axis represents the -log10 of the p-value derived from a t-test. P-values here are not corrected for multiple comparisons given the low number of samples included in the analysis. Dashed lines represent the typical thresholds used in differential gene expression to define significance (1 and -1 for the log2FoldChange and -log10(0.05) for the p-value). Genes in the top-right corner (green) are overexpressed in HLA-DR+ areas compared to HLA-DR- areas while genes in the top-left corner (red) are overexpressed in HLA-DR- areas. The gene names for the top 10 most significant genes are also included in their respective position. Differential gene expression of different HLA-DR genes are also included among the gene names. **(B)** Pathway analysis of HLA-DR+ areas compared to HLA-DR- areas. Interesting pathways from the top 30 up-regulated pathways are included.

To further corroborate and further expand the previous findings, we also performed a transcriptome analysis of micro dissected samples comparing Pos and NegInPos areas of selected cases with adequate RNA quality of the data set with frozen samples. From the 55,401 genes included in the analysis, we found 162 genes significantly overexpressed and 66 genes significantly under expressed (abs(logFC)>1, p-value<0,05) (**Figure 6A**, **Supplementary Table 5**). We identified the gene functions of the most expressed genes using the Genecards and Uniprot database (40, 41). The overexpressed genes in HLA-DR+ versus HLA-DR- areas were divided into 8 subgroups: (1) HLA class II and related genes (HLA-DPB1, HLA-DRB1, HLA-DPA1, CIITA, CTSS), (2) cytokines, chemokines and cell signaling receptors (TGFB1, TLR10, ZFP36, CXCL14, KIR2DL4, TNFSF15), (3) T and NK cell function related genes (KIR2DL4, CYTIP, TIM3/HAVCR2, FASLG, SLAMF7, BHLHE41, LGALS9), (4) B cell function related genes (BANK1, IGHM), (5) myeloid and monocyte related genes (MNDA, APOBEC3A), (6) cell growth and differentiation related genes (ST14, SPINT2, PRKCB, JUNB, MIXL1, ADIRF, RELB), (7) cell structure, motility and metabolic genes (APOL1, SNCG, SYTL3, CAPG, STAC3, MYOM1, DSP, DSC3, TINAGL1, BVES, FMO2, PLA2G4A, ALOX5) and (8) cell cycle related genes (RASSF2, RASSF6, WIF1, JUNB). The overexpression of several HLA class II genes of group 1 served as an internal control, confirming the correct location of the laser-micro dissected areas. The most interesting groups for our study are number 2, 3, 4, and 5, picturing a mixed inflammatory microenvironment including B cells, T cells, NK cells and monocytes. In all these groups we could distinguish genes exerting both anti-inflammatory roles (TGFB1, TLR10, ZFP36, CYTIP, TIM3/HAVCR2) as well as genes with pro-inflammatory and activating roles (CXCL14, KIR2DL4, KIR2DL4, FASLG, SLAMF7, BANK1). Moreover, genes that inhibit angiogenesis were also present (TNFSF15, CXCL14). In addition to TIM3/HAVCR2 overexpression, also LGALS9, encoding galectin-9, a main ligand of TIM3, was found to be overexpressed. Interestingly, the genes in the myeloid-related group (number 5) are mainly IFN-induced genes. In addition, we checked the expression of specific genes associated with immunosuppression/immune checkpoints that were not included in the MILAN panel, in particular PD-L1/CD274, IDO1 and CTLA4. Both PD-L1 and IDO1 were close to the significance threshold set for this study (PD-L1: p-value=0,067; IDO1: p-value=0,051). Instead, we did not find a significant differential expression for CTLA4. Finally, among the significantly under expressed genes we identified genes involved in more general cell functions such as cell cycle regulation and metabolism but no specific immune-related genes. The functions of the genes listed in this paragraph are further discussed in **Supplementary Table 6**.

In addition, we selected the 30 transcription factors predicted to be the most differentially active between Pos and NegInPos areas and divided them in 7 groups (**Supplementary Data 4**): (1) NFKB-signaling related transcription factors (RELA, RELB, NFKB1, LYL1), (2) IFNγ-signaling related transcription factors (STAT1, STAT2, USF1, IRF1, RFXANK, RFXAP, RFX5), (3) immune cell function-related transcription factors related to T cell (TBX21/T-bet), B cell (PAX5, POU2F2), both T and B cell (BATF, IKZF1) and more various immune cell (IRF4, SPI1, SPIB), (4) cell growth and differentiation-related transcription factors (FOS, JUN, JUND, SMAD3, ELF3, GRHL2, KLF5, SP1, ETS1, ERG) and (5) a transcription factor that is in normal circumstances restricted to ovarian tissue, that will not further be discussed (FOXL2). Also the transcription factor analysis supported the idea of a mixed immune microenvironment in the HLA-DR positive areas, with predominant IFNγ signature. The functions of the transcription factors listed in this paragraph are further discussed in **Supplementary Table 7**.

Finally, we performed pathway analysis and demonstrated an upregulation of multiple interesting biological pathways primarily involving the immune system function. In **Supplementary Table 8**, all the 2119 pathways included in the database used for pathways analysis are shown. Among these, 332 pathways were significantly upregulated in the HLA-DR positive areas compared to the HLA-DR negative areas. Among these, we looked for pathways that were relevant for immune-related processes and disregarded those not adding any relevant information to our study because they were linked to general biologic pathways (**Figure 6B**, **Supplementary Table 8**). Interestingly, we found again most of these pathways to be involved in B cell activation, NK and T cell functions (both helper and cytotoxic), plus upregulation of pathways involving dendritic cells and antigen presentation and of the PD-1 signaling pathway. From a cytokine point of view, the IFNγ and the IL-12 pathway were predicted to be the most active. Since some of the upregulated pathways show a significant number of overlapping genes, crosstalk between these pathways will definitely be present. The gene signatures of the pathways implicated in IFNγ (REACTOME_REGULATION_OF_IFNG_SIGNALING, BIOCARTA_IFNG_PATHWAY, PID_IFNG_PATHWAY) and IL-12/IL4 signaling (PID_IL12_2PATHWAY, BIOCARTA_NO2IL12_PATHWAY, PID_IL12_STAT4_PATHWAY, BIOCARTA_IL12_PATHWAY, PID_IL4_2PATHWAY), in particular, showed some overlapping genes (**Supplementary Figure 4**). Some of these were significantly overexpressed in our gene expression analysis in HLA-DR positive areas. In the IFNγ pathway, the only significantly overexpressed gene was IRF1, a downstream regulator of IFN-signaling that is rapidly induced by IFN-α, IFN-β and IFN-γ, and regulates the transcription of several IFN-γ-induced genes (20). In the case of IFN-γ-stimulation, this gene, together with USF1 cooperate in the STAT1-mediated transcription of CIITA, the master regulator of MHC II transcription (20). Concerning the IL12-pathway, CD247, FASLG, HLA-DRA, IL2RB and RELB were significantly overexpressed. CD247 encodes the protein T-cell receptor zeta, which is a subunit of the T-cell receptor-CD3 complex. The zeta chain plays an important role in coupling antigen recognition to several intracellular signal-transduction pathways and thus plays an essential role in the adaptive immune system. FASLG is the gene that encodes the protein FAS ligand, a membrane anchored protein of the TNF family that is present on activated T cells and NK cells and is essential for their cytotoxic function and T cell homeostasis. HLA-DRA encodes the alpha-subunit of HLA-DR, and is thus important for antigen presentation. IL2RB encodes the beta-subunit of the IL-2 receptor that plays a role in CD8+ T cell and NK cell mediated immune responses (42). RELB encodes a transcription factor that is involved in the alternative pathway of NFκB signaling, stimulated by a small number of TNF receptor superfamily members (such as CD40) (43). Finally, in the IL4 pathway the differential gene expression analysis showed that COL1A1, DOK2 and SOCS3, of which only SOCS3 is interesting enough to discuss. It encodes for a STAT-induced STAT inhibitor that suppresses cytokine signaling. Its expression is induced by IL6, IL10 and IFNG. This protein can inhibit the activity of JAK2 kinase, another gene in common between the IFNγ and the IL12 pathways (44).

### Cytokine Expression Profiling Suggests a Germinal Center-Like Environment in HLA-DR+ Areas

**Figure 7:**
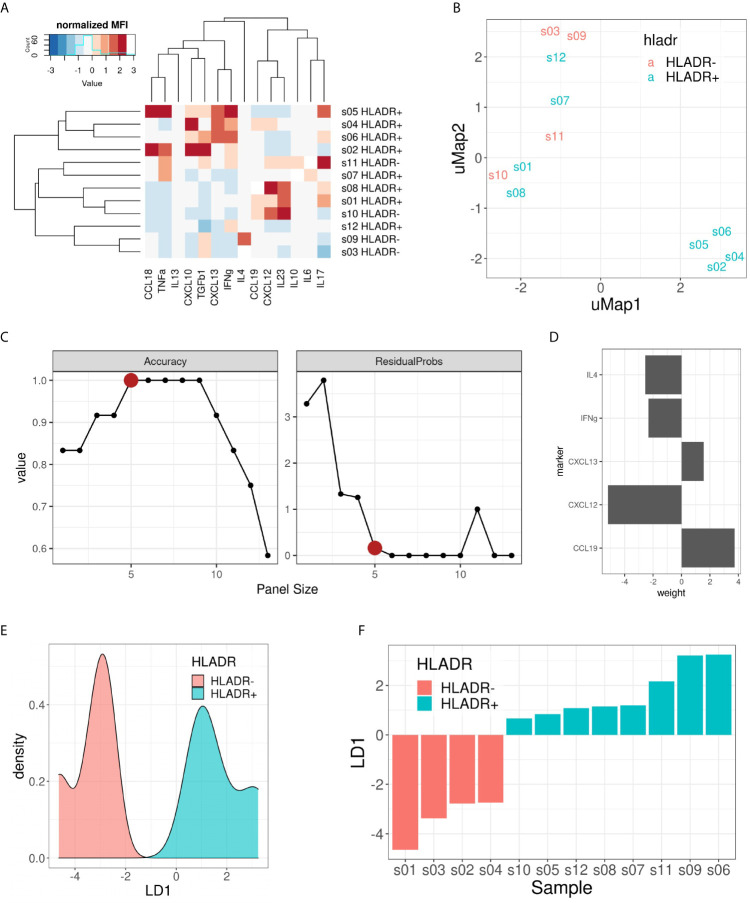
Multiplex ELISA assay. **(A)** Heat map representing the normalized (z-score) mean fluorescence intensity (MFI) of the 14 measured cytokines (columns) in the 12 samples included (rows). Both rows and columns are sorted based on hierarchical clustering. **(B)** uMap representing the partial separation of HLA-DR+ and HLA-DR- samples by unsupervised dimensionality reduction. **(C)** Pareto front representing model accuracy (left) and residual probability (right) of the best LDA model for each panel size (x-axis). Elbow criterion identified a panel size of 5 markers as the optimal one. **(D)** Weights of the 5 included cytokines for the optimal LDA model: CCL19, CXCL12, CXCL13, IFNγ and IL4. **(E)** Density plot on LD1 for the included samples. **(F)** Bar plot showing the separation of HLA-DR+ and HLA-DR- samples following the predictions made with the 5 cytokine panel.

Finally, we investigated which cytokines predominantly drive the composition of the tumor microenvironment in HLA-DR+ metastases. Therefore, we performed a customized Multiplex ELISA panel for the Luminex Flexmap 3D including IFNγ, IL6, IL10, TNFα, IL4, CXCL10, IL17, IL13, CCL18, TGFβ, IL23, CXCL13, CXCL12, and CCL19 comparing HLA-DR+ and entirely HLA-DR/- samples. Because sufficient material was needed to measure robust cytokine levels, we could not perform laser-assisted microdissection, but rather compared the overarching groups. The normalized (z-score) Mean Fluorescence Intensity (MFI) values of the different cytokines in each sample are summarized in **Figure 7A**. Initially, we explored the information content of these 14 markers using unsupervised dimensionality reduction (uMap). Unsupervised clustering separated only partially the HLA-DR+ and HLA-DR- cases (**Figure 7B**). This means that we had some informative markers that allowed us to distinguish HLA-DR+ from HLA-DR- melanomas, and uninformative markers that an unsupervised analysis cannot dismiss. In order to find the optimal discriminative panel, we trained Linear Discriminant Analysis (LDA) models as described in the methods, which identified a panel of 5 cytokines as the optimal panel (**Figure 7C**). These 5 markers included IFNγ, IL4, and the three germinal center cytokines CCL19, CXCL12 and CXCL13 (**Figure 7D**), highlighting a germinal center-like microenvironment in HLA-DR+ tumors. This limited 5-plex cytokine panel separated completely the melanoma metastases expressing HLA-DR from ones completely negative for HLA-DR (**Figures 7E, F**).

## Discussion

The MHC II complex is one of the main routes for antigen presentation and immune system activation, yet expression by melanoma cells is associated with a controversial role in literature, being described as an unfavorable prognostic factor in some studies and with longer survival in others (18, 24–28). Recently, MHC II expression, HLA-DR in particular, has also been correlated with response to anti-PD-1 therapy (13–16), nevertheless little is known about the biology behind this finding. To investigate the inflammatory microenvironment in HLA-DR positive melanoma, we first characterized at single cell level the immune microenvironment in HLA-DR positive and negative areas, then we investigated the upregulated genes and pathways in these areas and finally we confirmed the hypothesis generated by the first two levels of analysis by determining which cytokines are determinant in driving HLA-DR expression in melanoma.

Using MILAN, we identified in HLA-DR positive tumors a higher variety of inflammatory cell types compared to negative areas in the same tumor, where in particular a very low amount of B cells, cDC1, M1-like macrophages and TFH were present. This finding was also supported by the transcriptomic analysis that depicted a mixed immune microenvironment in the HLA-DR positive areas in comparison with HLA-DR negative areas, with overexpression of several genes, predicted upregulation of multiple transcription factors and activation of pathways linked to an increased presence of T cells, B cells and monocytes. Yet, comparing HLA-DR positive areas with HLA-DR negative tumors, the former showed a similar degree of variety in the inflammatory subtypes present, suggesting us that in HLA-DR positive tumors the HLA-DR positive areas will have the function of attract and concentrate most of the inflammatory cells infiltrating the tumor, pauperizing the HLA-DR negative areas instead.

Concentration and attraction of inflammatory cells is usually a feature of primary and secondary lymphoid organs, where a precise loco regional organization of lymphoid and myeloid cells is also present. It was therefore surprising for us to find in HLA-DR positive areas small communities of mixed T and B cells, in particular with Treg-TFH, TFH-Th and Tcy-BC_GerminalCenter interactions. Moreover, additional upregulated genes and pathways pointed at the presence of enhanced antigen presentation, B cell activation and B cell-specific processes. In particular, the *Bystander B cell pathway* regulates apoptosis of those B cells that are not activated by antigens, a process that usually takes place in the germinal centers of lymph nodes. Furthermore, transcription factor analysis showed upregulation of BATF and IRF4, that cooperatively regulate IL-4 production in TFH cells (45). Moreover, IRF4 is expected in plasma cells, B cell activation and germinal center B centrocytes (46). An immune microenvironment with these features could be comparable to a germinal center.

To provide more evidences about this, we went back to the single cell data and compared the cell-cell interaction profiles of negative tumors and HLA-DR positive areas in positive tumors with germinal centers/tertiary lymphoid structures, by performing a neighborhood analysis on our spatial single-cell data. Here, we found that our positive control, the germinal center itself, had a very specific interaction pattern involving a B cell community and a T cell community, and in comparison the close interactions in the positive areas also involved both T and B cells (BC_GerminalCenter-Tcy-Treg-TFH-Th) while in negative tumors only the T cell compartment showed significant cell-cell interactions (Tcy-TFH). Finally, we checked on a broad panel of cytokines which ones were in combination the most efficient in discriminating between negative and positive cases. The Luminex assay confirmed that the ones expressed in the germinal center microenvironment, together with IFNγ and IL4, best separated HLA-DR positive and negative cases.

This germinal-center-like microenvironment seems to be supported by the presence of pro-inflammatory cytokines. In particular, we found to be enriched in the HLA-DR positive microenvironment: IL12, at transcriptomic level, a cytokine that is secreted by phagocytic cells and stimulates the production of IFNγ and TNFα by NK cells and T cells, thereby enhancing their cytotoxic activity (47); and IFNγ both at transcriptomic and cytokine level, providing a nice and solid validation of our approach and confirming the well-known role of IFNγ in stimulating HLA-DR expression.

Nevertheless, the end result of this germinal center-like microenvironment appeared to have a dystrophic orientation towards immune suppression. First of all, overexpression of multiple immunomodulatory genes (TGFβ, HMOX1, TIM3) and pathways (*PD-1 pathway*) was found at transcriptomic level. In particular, TGFβ, inhibits the function of effector T cells and favors differentiation of naïve T cells into Tregs, and activity of the PD-1 pathway can lead to T cell exhaustion. This was already confirmed at single cell level, where not only overexpression of PD-1 was present in HLA-DR positive areas, but also higher levels of T cell exhaustion, significantly higher than in HLA-DR negative tumors and definitely more than in germinal centers, used as control for an area of generation of an efficient immune response.

Additional evidence points towards an hyper stimulation of the Tcy as the possible explanation for this exhaustion and immunosuppression enhancement in HLA-DR positive areas. Specifically, HLA-DR positive areas were found to be enriched in antigen-presenting cells (cDC1 and M1-like macrophages) at single cell level and associated with an enhanced activity of pathways linked to antigen presentation and dendritic cell functions in the transcriptomic analysis. Besides that, M1-like macrophages are found both in negative and positive tumors to be close neighbors of the melanoma cells. Though, while in negative tumors they are strongly close to HLA-DR negative melanoma cells, in positive tumors they have a preferential strong interaction with HLA-DR positive cells, and this may represent an overstimulating/confounding microenvironment in terms of antigen presentation leading to exhaustion and/or to a immunosuppressive shift in the microenvironment as a control mechanism to hyper immunity.

Finally, the high levels of PD-1 expression in these areas could also explain why anti-PD-1 therapy would be more efficient in HLA-DR positive tumors. In addition, the earlier described pan-tumor T-cell inflamed gene expression signature correlating with clinical benefit to anti-PD-1 treatment seems to partially overlap with the micro environmental changes specific for HLA-DR positive melanoma areas described here. This 18-gene immune panel contains among others CIITA, STAT1, HLA-DRA, CXCL13 and IFNγ (10). Although being described as an immune-specific signature, based on our findings a similar gene expression profile is to be expected in HLA-DR positive melanoma and hence could partially explain the high efficacy rate of checkpoint blockade in these patients. In addition, we could observe, in line with others (13, 39), higher PD-L1 expression in HLA-DR+ melanoma compared to HLA-DR- melanoma. Johnson and colleagues previously described a higher PD-1/PD-L1 interaction score to be predictive for response to immunotherapy, not considering the underlying type of cell-cell interaction or the cell types expressing these markers and independent of HLA-DR expression by the melanoma cells (14). Our findings, showing higher PD-1 expression levels in the immune cells in the tumor areas containing HLA-DR+ melanoma cells in addition to higher PD-L1 expression in the HLA-DR+ melanoma cells themselves, highlight a similar PD-1/PD-L1 proximity, potentially driven by HLA-DR expression in the melanoma cells that could explain the predictive potential of the expression of HLA-DR.

Despite its novelty, our study is not exempt of limitations. First of all, the number of patients with HLA-DR+ and HLA-DR- melanoma included in our analysis is rather limited. Although the aim of the study was to investigate the specific microenvironment of HLA-DR expressing melanomas to elucidate an explanation for the predictive potential of HLA-DR for response to immunotherapy observed by others rather than producing a patient classifier, the validity of our findings would be certified if applicable on a larger patient cohort. Nonetheless, the main conclusion of the germinal center-like microenvironment in HLA-DR + melanoma is corroborated using multi-omics applied on different (small) patient cohorts. In addition, the predictive potential of HLA-DR expression for response to immunotherapy has been described in literature by others (13, 16, 48). Independent of this observation, tumor microenvironmental analysis in melanoma and even more so in HLA-DR+ melanoma has not been given sufficient attention within literature. Driven by these 2 aforementioned observations, in our analysis we had the intent to explore the local microenvironment of HLA-DR expressing melanoma and particularly what is different from the tumor microenvironment of melanoma cells that do not express HLA-DR, and by doing so potentially provide a first insight on why there is an improved response to immunotherapy. Hence, because our samples were selected using only the expression of HLA-DR in melanoma metastases without considering treatment history prior or after sampling during this selection, as it was not the primary objective of our study, we cannot correlate our findings with response to therapy. Therefore, it remains unclear and speculative whether our findings in the specific local microenvironment are in fact the reason why these patients tend to respond better to immunotherapy. Moreover, in a small subset of pretreatment biopsy or resection specimens from 30 patient treated with anti-PD-1 or anti-PD-L1, objective response rate was significantly higher in the HLA-DR + subset (79% versus 38%), yet still lacking response in 21% of the patients (13). Although further validation of these findings is needed in a bigger patient cohort, micro environmental differences between responding HLA-DR+ melanoma and non-responding HLA-DR+ melanoma still remain to be elucidated.

In conclusion, we found that HLA-DR positive areas in melanoma attract and concentrate the anti-tumor immune cell infiltration creating a germinal center-like microenvironment, though presenting dystrophic features. This microenvironment in fact seems to lead to an exhausted microenvironment through hyperactivity of the antigen presentation pathways, nevertheless representing a fertile ground for a better efficacy of anti-PD1 inhibitors.

## Data Availability Statement

The data presented in the study are deposited in the European Nucleotide Archive (ENA) repository, accession number PRJEB41749. 

## Ethics Statement

Ethical approval was obtained from the Ethical Committee/IRB OG032 of the University Hospital of Leuven. After the approval, the study was identified with the number S57266. According to the Clinical Trial regalement no informed consent was needed due to the use of post-diagnostic left-over material. Written informed consent for participation was not required for this study in accordance with the national legislation and the institutional requirements. Written informed consent was not obtained from the individual(s) for the publication of any potentially identifiable images or data included in this article.

## Author Contributions

LG performed microdissection, RNA extraction, transcriptomics interpretation and wrote the paper. YH collected clinical data, reviewed the processed images, participated in the MILAN analysis, prepared the images and wrote the paper. GM participated in the data analysis, prepared images and reviewed the paper. ZK performed the RNAseq data processing and analysis. MB reviewed and processed the raw images. JW performed the RNAseq data analysis. LM supervised LG in the experiments and reviewed the paper. AM supervised FB in the preparation of the samples and the design of the Luminex. VP executed part of the Luminex. JR executed part of the Luminex. LA provided reagents, machines and guidance for the Luminex. GC executed the stainings according to the MILAN protocol and provided guidance for MILAN. OB collected clinical data, gave clinical guidance and reviewed the paper. JO designed the project. FS gave critical insight to the project and reviewed the paper. AA coordinated all the dry lab analysis, performing the transcription factor and pathways analysis, the Luminex analysis, all the MILAN downstream analysis and wrote the paper FB designed the project, performed part of the wet lab analysis, guided the first co-authors, interpreted the results, and wrote the paper. All authors contributed to the article and approved the submitted version.

## Funding

This work was funded by the MEL-PLEX research training program (‘Exploiting MELanoma disease comPLEXity to address European research training needs in translational cancer systems biology and cancer systems medicine’, Grant agreement no: 642295, MSCA-ITN-2014-ETN, Project Horizon 2020, in the framework of the MARIE SKŁODOWSKA-CURIE ACTIONS), the SyMBioSys research training programme (‘Systematic Modeling of Biological Systems’), grant agreement no: 675585, MSCA-ITN-2015-ETN, Project Horizon 2020, in the framework of the MARIE SKŁODOWSKA-CURIE ACTIONS, and the Regione Lombardia POR FESR 2014-2020, Call HUB Ricerca ed Innovazione: ImmunHUB to GC.

## Conflict of Interest

Authors AM, VP, JR, and LA were employed by the company ProtATonce Ltd. 

The remaining authors declare that the research was conducted in the absence of any commercial or financial relationships that could be construed as a potential conflict of interest.

## Supplementary Material

Supplementary Figure 1 Phenotypic identification. **(A)** Expression fingerprints. Average expression profile of the identified cell phenotypes after clustering and manual annotation (see *Methods*). M indicates the mean expression of a given marker for a given cell phenotype. **(B)** Histogram showing the distribution of HLA-DR expression in melanoma cells (asinh transformed) used for their manual gating. A threshold of 2 was selected to separate HLA-DR positive from HLA-DR negative melanoma cells. **(C)** Histogram showing the distribution of PD1 expression in CD3+CD4+ T cells (asinh transformed) used for manual gating. A threshold of 2 was selected to separate T Follicular Helpers (TFH, PD1+) from wild-type T Helpers (TH, PD1-). **(D)** 2D histogram showing the distribution of BCL2 and BCL6 in B cells (asinh transformed) used for their manual gating. A threshold of 2 was selected in both markers to separate germinal center B cells (BCL6+/BCL2-), early germinal center B cells (BCL6+/BCL2+) and B cells not further specified (BCL6-/BCL2- or BCL6-/BCL2+).

Supplementary Figure 2 Cell proportion single cell composition of HLA-DR+ areas, HLA-DR- areas in positive tumours, HLA-DR- tumours, germinal centers and tertiary lymphoid structures. Boxplots indicating the relative proportion of different cell-types in the different micro dissected areas, from left to right: ‘GC’ (Germinal centre from reactive lymph nodes; orange), TLS (germinal centers from tertiary lymphoid structures, ochre), ‘NegTum’ (Tumour border of HLA-DR negative tumours, green), ‘ NeginPos’ (HLA-DR negative area of HLA-DR positive tumours, blue) and ‘Pos’ (HLA-DR positive area of HLA-DR positive tumours, pink). Blood_V= Blood vessel; cDC2= Classical dendritic cell type II; Th= T helper cell; TFH= T follicular helper cell; Treg= Regulatory t cell; Lymph_V= Lymphatic vessel; PC= Plasma cell. Significance levels indicate: * p-value ≤ 0.05, ** p-value ≤ 0.01, *** p-value ≤ 0.001, **** p-value ≤ 0.0001. P-values are derived from Wilcoxon tests (no fdr corrected).

Supplementary Figure 3 Cell density single cell composition of HLA-DR+ areas, HLA-DR- areas in positive tumours, HLA-DR- tumours, germinal centers and tertiary lymphoid structures. Boxplots indicating the cell density (cells/mm²) of different cell-types in the different micro dissected areas, from left to right: ‘GC’ (Germinal centre from reactive lymph nodes; orange), TLS (germinal centers from tertiary lymphoid structures, ochre), ‘NegTum’ (Tumour border of HLA-DR negative tumours, green), ‘ NeginPos’ (HLA-DR negative area of HLA-DR positive tumours, blue) and ‘Pos’ (HLA-DR positive area of HLA-DR positive tumours, pink). BC= B cell not further specified; BC_EarlyGerminalCenter= Early germinal center B cell; BC_GerminalCenter= Germinal center B cell; PC= Plasma cell; Th= T helper cell; Treg= Regulatory t cell; TFH= T follicular helper cell; Tcy= Cytotoxic t cell; NK= Natural killer cell; cDC1= Classical dendritic cell type I; cDC2= Classical dendritic cell type II; fDC= Follicular dendritic cell; pDC= Plasmacytoid dendritic cell; macrop= Macrophage; macrop_CD163= CD163 positive macrophage; HLApos_mel= HLA-DR positive melanoma cell; HLADRneg_mel= HLA-DR negative melanoma cell; Blood_V= Blood vessel; HEV= High endothelial venule; Lymph_V= Lymphatic vessel. Significance levels indicate: * p-value ≤ 0.05, ** p-value ≤ 0.01, *** p-value ≤ 0.001, **** p-value ≤ 0.0001. P-values are derived from Wilcoxon tests (no fdr corrected).

Supplementary Figure 4 Volcano plots showing the differential expression between HLA-DR+ and HLA-DR- areas of genes included in different IGNy and IL-4/IL-12 pathways obtained from the Molecular Signatures Database. **(A)** BIOCARTA_IL12_PATHWAY, **(B)** PID_IFNG_PATHWAY, **(C)** BIOCARTA_IFNG_PATHWAY, **(D)** PID_IL12_STAT4_PATHWAY, **(E)** REACTOME_REGULATION_OF_IFNG_SIGNALING, **(F)** BIOCARTA_NO2IL12_PATHWAY, **(G)** PID_IL12_2PATHWAY, **(H)** PID_IL4_2PATHWAY. The x-axis represents the log2 of the fold change in expression between HLA-DR positive and HLA-DR negative areas while the y-axis represents the -log10 of the p-value of a t-test comparing the expression values of these areas.
