# Supplementary figures and images for: A Multi-Omics Analysis of Metastatic Melanoma Identifies a Germinal Center-Like Tumor Microenvironment in HLA-DR-Positive Tumor Areas
Source: Front Oncol. 2021 Mar 25;11:636057. doi: 10.3389/fonc.2021.636057 (PMC8029980; doi:10.3389/fonc.2021.636057)

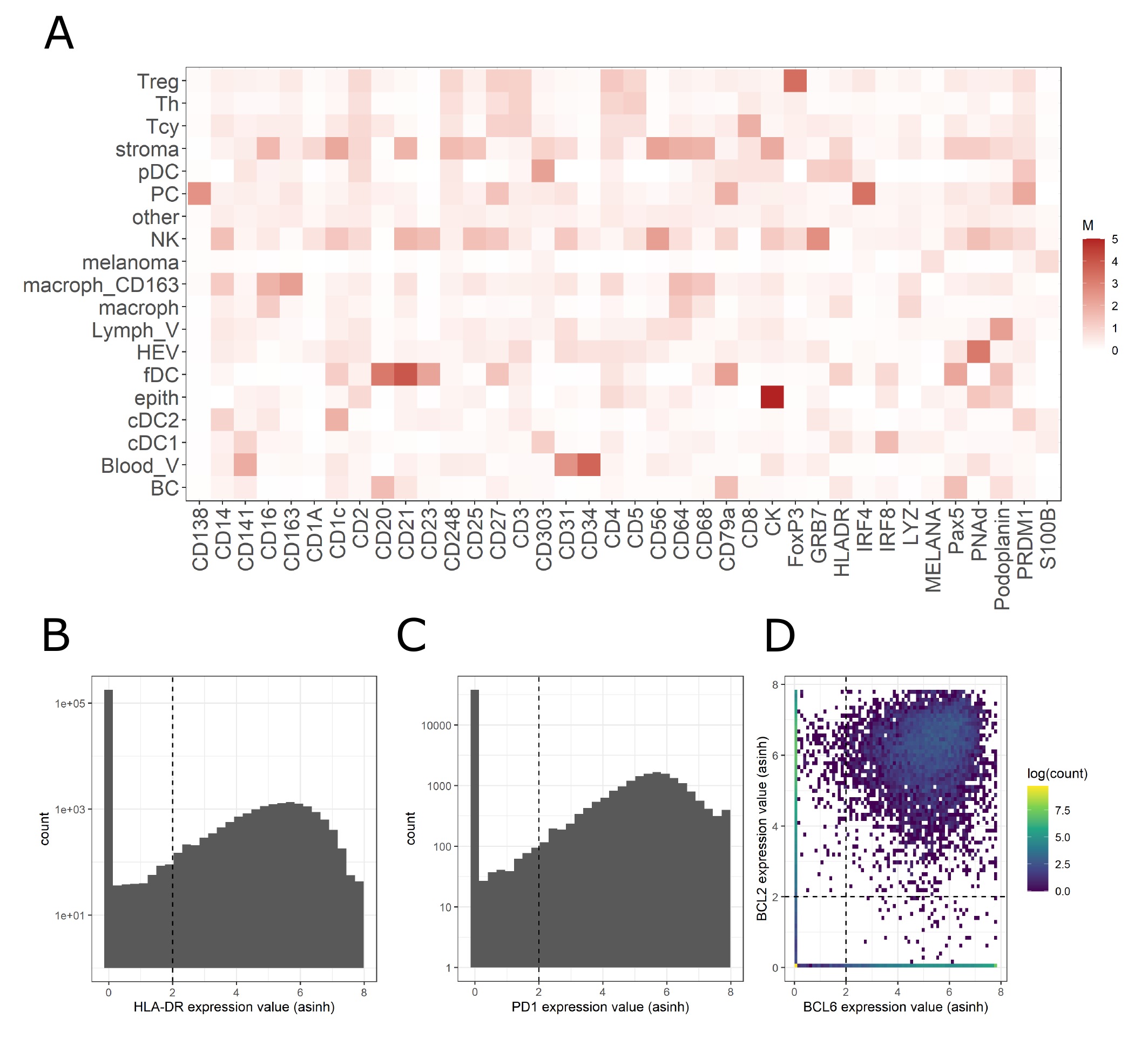

Supplement: Supplementary Figure 1 — Phenotypic identification. (A) Expression fingerprints. Average expression profile of the identified cell phenotypes after clustering and manual annotation (see Methods). M indicates the mean expression of a given marker for a given cell phenotype. (B) Histogram showing the distribution of HLA-DR expression in melanoma cells (asinh transformed) used for their manual gating. A threshold of 2 was selected to separate HLA-DR positive from HLA-DR negative melanoma cells. (C) Histogram showing the distribution of PD1 expression in CD3+CD4+ T cells (asinh transformed) used for manual gating. A threshold of 2 was selected to separate T Follicular Helpers (TFH, PD1+) from wild-type T Helpers (TH, PD1-). (D) 2D histogram showing the distribution of BCL2 and BCL6 in B cells (asinh transformed) used for their manual gating. A threshold of 2 was selected in both markers to separate germinal center B cells (BCL6+/BCL2-), early germinal center B cells (BCL6+/BCL2+) and B cells not further specified (BCL6-/BCL2- or BCL6-/BCL2+). [file DataSheet_1.zip › Supplementary Figure 1.JPEG]

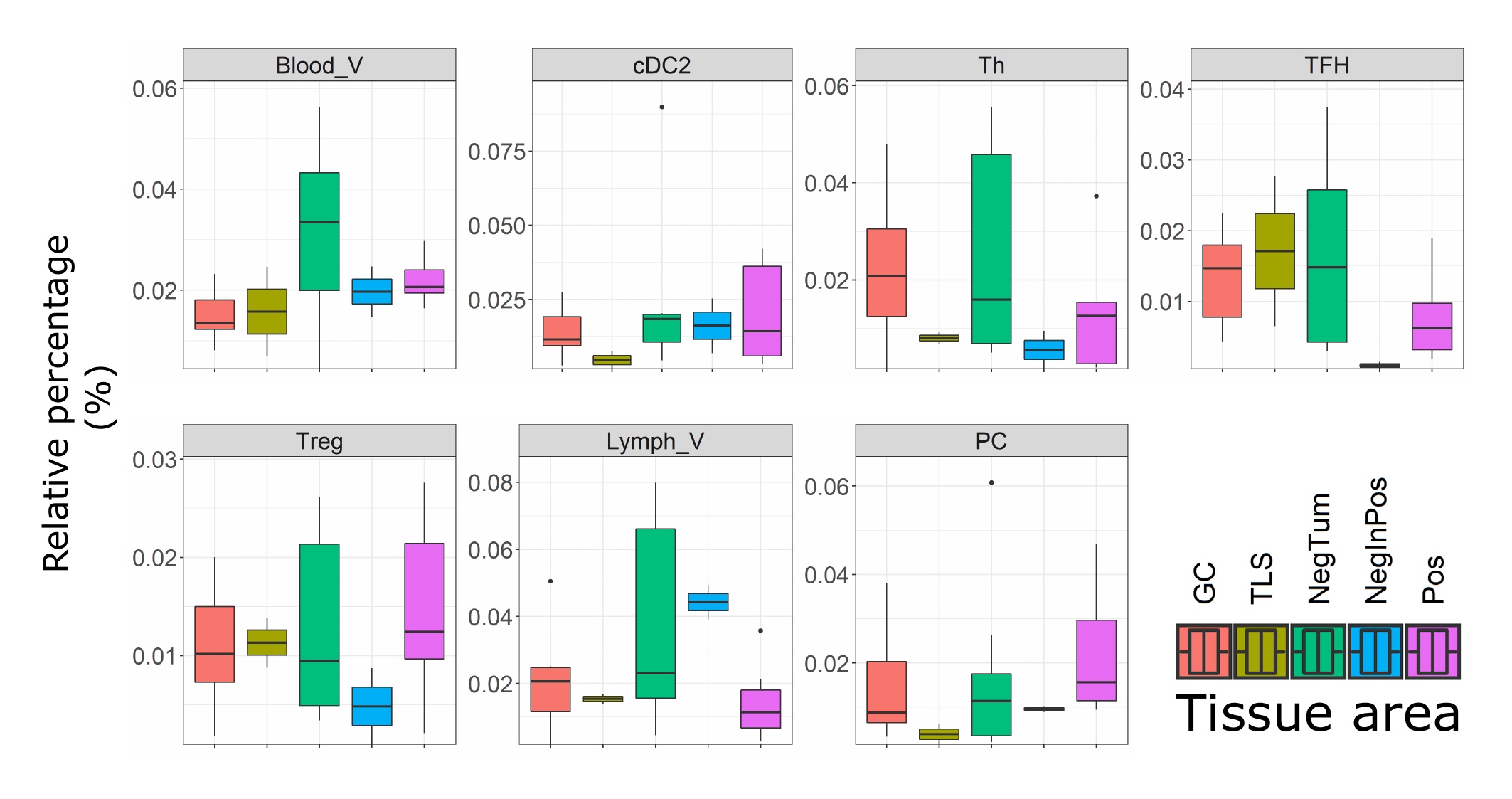

Supplement: Supplementary Figure 1 — Phenotypic identification. (A) Expression fingerprints. Average expression profile of the identified cell phenotypes after clustering and manual annotation (see Methods). M indicates the mean expression of a given marker for a given cell phenotype. (B) Histogram showing the distribution of HLA-DR expression in melanoma cells (asinh transformed) used for their manual gating. A threshold of 2 was selected to separate HLA-DR positive from HLA-DR negative melanoma cells. (C) Histogram showing the distribution of PD1 expression in CD3+CD4+ T cells (asinh transformed) used for manual gating. A threshold of 2 was selected to separate T Follicular Helpers (TFH, PD1+) from wild-type T Helpers (TH, PD1-). (D) 2D histogram showing the distribution of BCL2 and BCL6 in B cells (asinh transformed) used for their manual gating. A threshold of 2 was selected in both markers to separate germinal center B cells (BCL6+/BCL2-), early germinal center B cells (BCL6+/BCL2+) and B cells not further specified (BCL6-/BCL2- or BCL6-/BCL2+). [file DataSheet_1.zip › Supplementary Figure 2.JPEG]

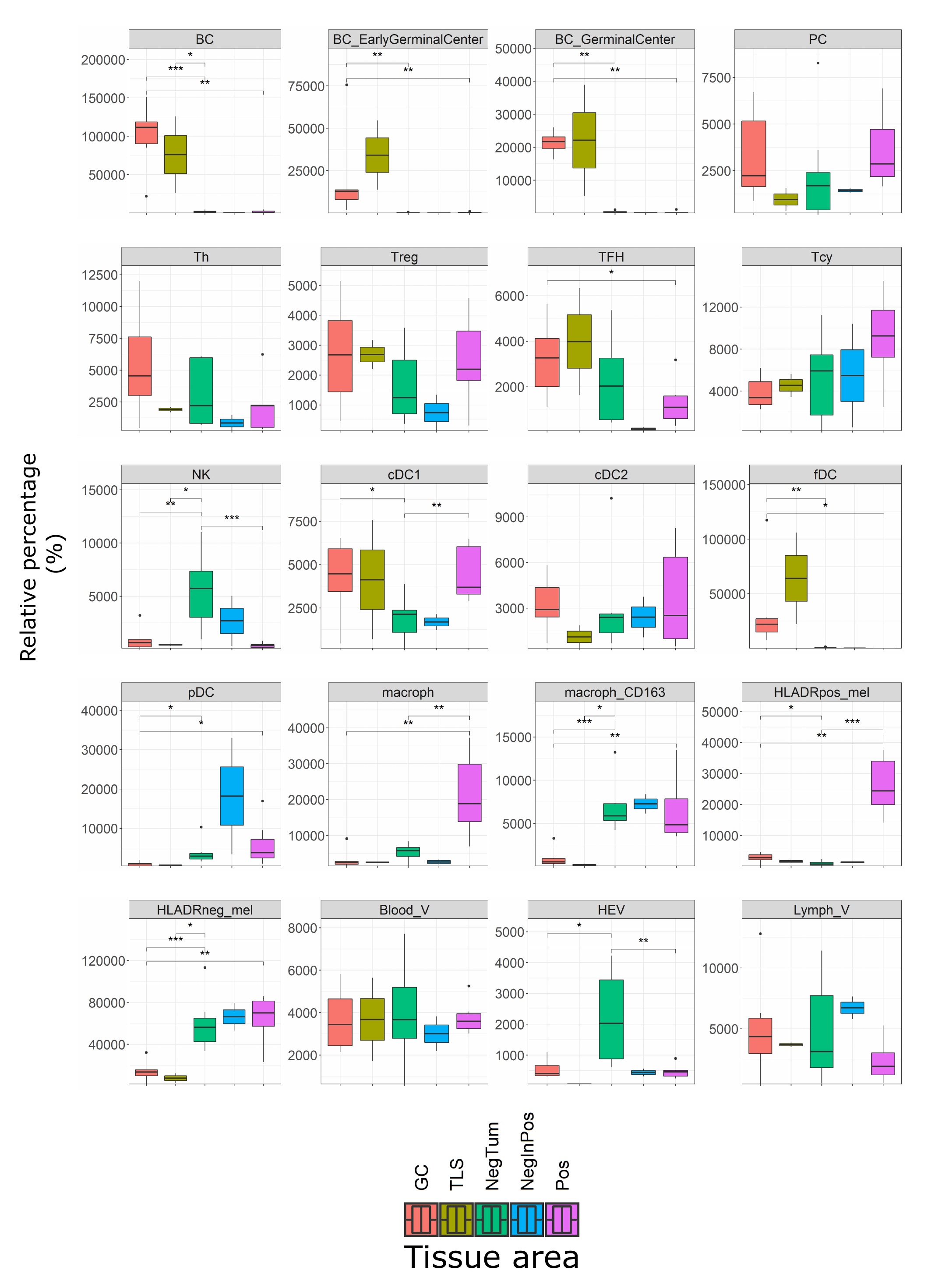

Supplement: Supplementary Figure 1 — Phenotypic identification. (A) Expression fingerprints. Average expression profile of the identified cell phenotypes after clustering and manual annotation (see Methods). M indicates the mean expression of a given marker for a given cell phenotype. (B) Histogram showing the distribution of HLA-DR expression in melanoma cells (asinh transformed) used for their manual gating. A threshold of 2 was selected to separate HLA-DR positive from HLA-DR negative melanoma cells. (C) Histogram showing the distribution of PD1 expression in CD3+CD4+ T cells (asinh transformed) used for manual gating. A threshold of 2 was selected to separate T Follicular Helpers (TFH, PD1+) from wild-type T Helpers (TH, PD1-). (D) 2D histogram showing the distribution of BCL2 and BCL6 in B cells (asinh transformed) used for their manual gating. A threshold of 2 was selected in both markers to separate germinal center B cells (BCL6+/BCL2-), early germinal center B cells (BCL6+/BCL2+) and B cells not further specified (BCL6-/BCL2- or BCL6-/BCL2+). [file DataSheet_1.zip › Supplementary Figure 3.JPEG]

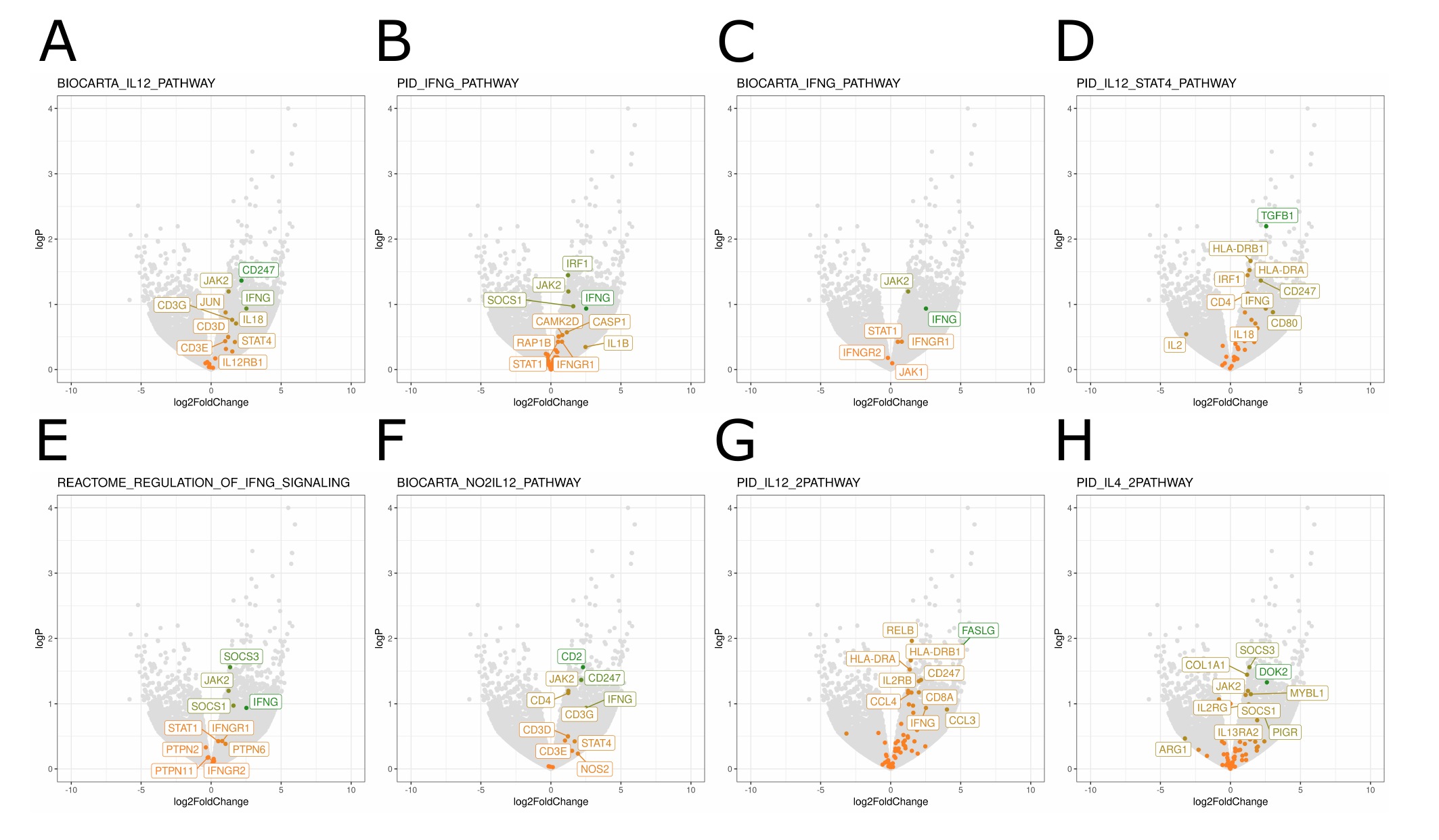

Supplement: Supplementary Figure 1 — Phenotypic identification. (A) Expression fingerprints. Average expression profile of the identified cell phenotypes after clustering and manual annotation (see Methods). M indicates the mean expression of a given marker for a given cell phenotype. (B) Histogram showing the distribution of HLA-DR expression in melanoma cells (asinh transformed) used for their manual gating. A threshold of 2 was selected to separate HLA-DR positive from HLA-DR negative melanoma cells. (C) Histogram showing the distribution of PD1 expression in CD3+CD4+ T cells (asinh transformed) used for manual gating. A threshold of 2 was selected to separate T Follicular Helpers (TFH, PD1+) from wild-type T Helpers (TH, PD1-). (D) 2D histogram showing the distribution of BCL2 and BCL6 in B cells (asinh transformed) used for their manual gating. A threshold of 2 was selected in both markers to separate germinal center B cells (BCL6+/BCL2-), early germinal center B cells (BCL6+/BCL2+) and B cells not further specified (BCL6-/BCL2- or BCL6-/BCL2+). [file DataSheet_1.zip › Supplementary Figure 4.JPEG]
