# Supplementary material for: A Multi-Omics Analysis of Metastatic Melanoma Identifies a Germinal Center-Like Tumor Microenvironment in HLA-DR-Positive Tumor Areas
Source: Front Oncol. 2021 Mar 25;11:636057. doi: 10.3389/fonc.2021.636057 (PMC8029980; doi:10.3389/fonc.2021.636057)
Supplement: Supplementary file 2 [file Table_1.docx]

**Supplementary Table 1: Overview of clinical information.** HLA-DR positive or negative status for each analysis type as defined within the methods section; NMM = nodular malignant melanoma, SSMM = superficial spreading malignant melanoma, DMM = desmoplastic malignant melanoma, NVMM = naevoid malignant melanoma, ALM = acral lentiginous melanoma; N/A: not applicable.

| **Patient ID** | **Analysis** | **HLA-DR status** | **Sample type** | **Gender** | **Age at sampling**  **(years)** | **Mutation status** | **Melanoma subtype of**  **primary lesion** |
| --- | --- | --- | --- | --- | --- | --- | --- |
| MEL1 | MILAN | negative | cutaneous metastasis | female | 67 | NRAS Q61R | NMM |
| MEL2 | MILAN | negative | intestinal metastasis | male | 35 | triple wild type | SSMM |
| MEL3 | MILAN | negative | subcutaneous metastasis | male | 44 | BRAF V600E | NVMM |
| MEL4 | MILAN | negative | lymph node metastasis | male | 42 | BRAF V600E | SSMM |
| MEL5 | MILAN | negative | subcutaneous metastasis | male | 78 | KIT | SSMM |
| MEL6 | MILAN | positive | cutaneous metastasis | female | 59 | BRAFV600E | SSMM |
| MEL7 | MILAN | positive | lymph node metastasis | male | 66 | NRAS Q61R | N/A |
| MEL8 | MILAN | positive | cutaneous metastasis | male | 64 | BRAF V600E | NMM |
| MEL9 | MILAN | positive | lymph node metastasis | female | 51 | BRAF V600E | NMM |
| MEL10 | RNAseq | positive | cutaneous metastasis | female | 72 | NRAS Q61R | NMM |
| MEL11 | RNAseq | positive | spleen metastasis | male | 62 | Unknown | SSMM |
| MEL12 | RNAseq | positive | intestinal metastasis | male | 52 | Unknown | SSMM |
| MEL13 | RNAseq | positive | bladder metastasis | female | 46 | BRAF V600E | Unknown |
| MEL14 | RNAseq | positive | intestinal metastasis | female | 67 | Unknown | Unknown |
| MEL15 | Luminex | positive | cutaneous metastasis | female | 73 | Unknown | Unknown |
| MEL16 | Luminex | positive | subcutaneous metastasis | female | 73 | Unknown | NMM |
| MEL17 | Luminex | positive | intestinal metastasis | male | 48 | Unknown | Unknown |
| MEL18 | Luminex | positive | subcutaneous metastasis | female | 45 | BRAF V600E | NMM |
| MEL19 | Luminex | positive | intestinal metastasis | male | 64 | Unknown | Unknown |
| MEL20 | Luminex | negative | intestinal metastasis | female | 49 | BRAF V600M | Unknown |
| MEL21 | Luminex | negative | cutaneous metastasis | female | 63 | BRAF V600E | ALM |
| MEL22 | Luminex | negative | cutaneous metastasis | female | 56 | Unknown | ALM |
| MEL23 | Luminex | negative | cutaneous metastasis | female | 58 | BRAF V600E | SSMM |
| GC1 | MILAN | N/A | lymph node | male | 54 | N/A | N/A |
| GC2 | MILAN | N/A | lymph node | female | 44 | N/A | N/A |
| GC3 | MILAN | N/A | lymph node | female | 44 | N/A | N/A |
| GC4 | MILAN | N/A | lymph node | male | 62 | N/A | N/A |
| TLS1 | MILAN | N/A | cutaneous metastasis | male | 60 | Unknown | Unknown |
