# Supplementary material for: A Multi-Omics Analysis of Metastatic Melanoma Identifies a Germinal Center-Like Tumor Microenvironment in HLA-DR-Positive Tumor Areas
Source: Front Oncol. 2021 Mar 25;11:636057. doi: 10.3389/fonc.2021.636057 (PMC8029980; doi:10.3389/fonc.2021.636057)
Supplement: Supplementary file 4 [file Table_3.docx]

| **Cell type** | **Cell subtype** | **GC** | **NegInPos** | **NegTum** | **Pos** | **TLS** |
| --- | --- | --- | --- | --- | --- | --- |
| B cell | Not further specified | 116 068 | 107 | 1 879 | 1 070 | 118 104 |
|  | Early germinal center | 10 450 | 21 | 235 | 289 | 16 955 |
|  | Germinal center | 21 442 | 43 | 364 | 126 | 36 269 |
| Plasma cell | N/A | 3 228 | 1 418 | 2 413 | 3 088 | 1 471 |
| T cell | T helper | 6 594 | 709 | 2 765 | 1 408 | 1 721 |
|  | Regulatory T cell | 3 143 | 601 | 1 832 | 2 197 | 2 276 |
|  | T Follicular Helper | 3 638 | 129 | 1 644 | 1 058 | 1 998 |
|  | Cytotoxic T cell | 4 103 | 4 318 | 5 747 | 8 423 | 3 608 |
| Natural Killer cell | N/A | 627 | 3 244 | 4 779 | 313 | 527 |
| Dendritic cell | Classical dendritic cell type I | 4 929 | 1 804 | 1 855 | 4 635 | 7 021 |
|  | Classical dendritic cell type II | 3 783 | 2 728 | 2 554 | 4 085 | 1 748 |
|  | Follicular dendritic cell | 20 778 | 107 | 522 | 81 | 28 860 |
|  | Plasmacytoid dendritic cell | 905 | 14 715 | 2 965 | 4 586 | 583 |
| Macrophage | Macrophage | 3 318 | 2 814 | 5 953 | 24 963 | 2 331 |
|  | CD163 positive macrophage | 959 | 7 003 | 7 103 | 5 896 | 111 |
| Melanoma | HLA-DR+ melanoma | 3 276 | 1 418 | 816 | 27 413 | 2 109 |
|  | HLA-DR- melanoma | 17 563 | 69 471 | 64 363 | 69 429 | 11 627 |
| Vasculature | Blood vessel | 3 753 | 2 814 | 4 098 | 3 809 | 2 026 |
|  | High Endothelial Venule | 513 | 408 | 1749 | 330 | 56 |
|  | Lymphatic vessel | 5 587 | 6 509 | 4 761 | 2 205 | 3 524 |
| Epithelial cell | N/A | 278 | 430 | 657 | 171 | 139 |
| Stromal cells | N/A | 646 | 5 993 | 2 982 | 4 980 | 416 |
| Other | N/A | 8 549 | 24 081 | 11 571 | 5 762 | 5 717 |

**Supplementary table 3: Cell density within different areas.** Overview of the cell density (cells/mm²) of all identified cell types in the MILAN analysis in the different areas. GC = Germinal centers from reactive lymph nodes; NegInPos = HLA-DR- areas in HLA-DR+ tumor; NegTum = Tumor border of HLA-DR- tumor; Pos = HLA-DR+ area in HLA-DR+ tumor; TLS = Tertiary lymphoid structure.
